# Outdoor Secondhand Smoke Exposure Around a Public Smoking Area: Formative Field Study Using Passive Wi-Fi Packet Sensing

**DOI:** 10.2196/90261

**Published:** 2026-05-05

**Authors:** Ryo Horiike, Kazuya Taira, Izumi Kondo, Motoyo Nawate, Harumi Bando

**Affiliations:** 1 Department of Nursing, School of Medicine, Nara Medical University, 88 Shijomachi, Kashihara, Nara, 634-0813, Japan, 81 744223051 ext 693313; 2 Center for Quantum Information and Quantum Biology, The University of Osaka, Toyonaka, Osaka, Japan; 3 Human Health Sciences, Graduate School of Medicine, Kyoto University, Kyoto, Kyoto, Japan

**Keywords:** tobacco, secondhand smoke, outdoor smoking, Wi-Fi, public health

## Abstract

**Background:**

Outdoor secondhand smoke (SHS) remains a public health concern, particularly around designated outdoor smoking areas where nonsmokers may pass through or remain nearby. Although prior studies have quantified outdoor SHS concentrations, fewer have examined how many people may be present within a plausible exposure setting. Estimating the exposure-opportunity level requires methods that are feasible, scalable, and minimally intrusive.

**Objective:**

This study aimed to evaluate the feasibility of using passive Wi-Fi packet sensing, calibrated with brief on-site observation, to estimate the number of smokers and passersby within a plausible SHS exposure range at a public outdoor smoking area in Japan.

**Methods:**

We conducted a formative field study at a designated outdoor smoking area at the Asia Pacific Trade Center in Osaka, Japan. A passive Wi-Fi packet sensor collected timestamps, anonymized device identifiers, organizationally unique identifiers, and received signal strength indicator (RSSI) values from October 13 to 29, 2023. The main analysis focused on October 28, 2023, a high-footfall event day selected for direct calibration. Episodes were classified using empirically derived RSSI thresholds, and class-specific calibration ratios were applied to estimate day-level counts.

**Results:**

Of 128,313 anonymized detections recorded on October 28, 90.3% (115,950/128,313) occurred during business hours. Among these, 8.6% (n=11,068) identifiers were detected more than once. Dwell time could be calculated for 1.4% (n=1817) of the identifiers, and 0.5% (n=659) eligible presence episodes remained after preprocessing. During a 30-minute validation window, smokers and passersby were counted manually within a 25-m radius. During the validation window, 6230 signal records formed 104 stays, with a mean stay duration of 9.89 (SD 7.89) minutes. During the validation window, direct observation recorded 14 smokers and 207 passersby within the 25-m radius. Applying the rule-based classification and calibration ratios to business hours data yielded estimated day totals of 262 smokers and 3907 passersby within the plausible SHS exposure range. Estimated smoker counts showed 2 peaks, around noon and 4 PM, whereas passerby volume peaked around midday. In an exploratory analysis, a random forest model using stay duration, mean RSSI, and RSSI variability achieved an accuracy of 0.95, sensitivity of 0.75, specificity of 0.97, and area under the receiver operating characteristic curve of 0.99.

**Conclusions:**

This formative field study suggests that passive Wi-Fi packet sensing, combined with brief on-site observation, can be used to estimate population-level exposure opportunity around an outdoor smoking area. The method identified substantial numbers of potentially exposed passersby in a high-footfall public setting. Although the findings are site specific and preliminary, they indicate that exposure-count metrics may complement concentration-based and survey-based SHS research. Further studies incorporating repeated validation, direct pollutant monitoring, and multiple sites are needed to refine the method and strengthen its usefulness for tobacco control and public health decision-making.

## Introduction

Secondhand smoke (SHS) remains an important and preventable cause of morbidity and mortality. Even brief exposure has been associated with acute endothelial dysfunction and adverse cardiovascular effects, and more recent evidence continues to support associations between SHS and multiple cardiovascular outcomes [1-3].

As comprehensive indoor smoke-free policies have expanded, exposure has shifted to outdoor and semiopen environments, where tobacco smoke may accumulate near active smokers and drift into adjacent spaces. Field measurements and systematic reviews have shown elevated PM2.5 concentrations in outdoor smoking environments, particularly in hospitality venues and partially enclosed settings, indicating that outdoor SHS cannot be assumed to be negligible [4-7]. Additional observational studies have also documented substantial outdoor SHS exposure in public environments, including locations frequented by minors, reinforcing concerns about routine exposure outside fully enclosed indoor settings [8-10].

A separate body of research has examined SHS exposure at the population level using survey data. These studies have shown that exposure varies by education, employment, income, and other sociodemographic characteristics, suggesting that SHS exposure is unevenly distributed across populations [11,12]. However, such studies generally rely on self-reported exposure or broad population indicators and do not quantify how many people are physically present within a plausible exposure zone around a specific outdoor smoking area.

In Japan, the 2020 revisions to the Health Promotion Act introduced national smoke-free requirements but retained exemptions such as designated smoking rooms and certain small venues. Population-based evaluations suggest that these partial measures have provided incomplete protection and that stronger, more comprehensive approaches remain warranted [13,14]. Nevertheless, evidence remains limited regarding the scale of potential SHS exposure around outdoor smoking areas in busy public environments, where large numbers of nonsmokers may pass through affected spaces even in the absence of direct pollutant monitoring.

At the same time, passive crowd sensing using wireless probe requests from personal devices has developed into a privacy-preserving and low-cost approach for estimating pedestrian flows without network association. Prior work has shown that anonymized Wi-Fi probe request logs can produce robust counts and temporal profiles in public spaces [15-18]. However, these approaches have largely been used for mobility, pedestrian flow, or retail analytics rather than for estimating tobacco-related exposure opportunity in real-world public health settings.

Against this background, we conducted a formative field study at a designated outdoor smoking area in a large commercial complex in Osaka, Japan. The aim of this study was to estimate the numbers of smokers and passersby present within a plausible SHS range during business hours on a high-footfall event day and to examine the feasibility of using passive Wi-Fi packet sensing, calibrated with brief on-site observation, to derive exposure-opportunity metrics relevant to public health practice and the management of smoking areas in public spaces.

## Methods

### Study Design and Setting

This formative field study was conducted at a designated outdoor smoking area located at the Asia Pacific Trade Center, a large commercial complex in Suminoe Ward, Osaka, Japan. The smoking area is adjacent to the Seaside Stage on the second floor of the O’s North building and consists of a fence and ashtray installed along the building facade. The fence is made of wire mesh and does not block airflow, and the area opens directly onto a major pedestrian corridor used by visitors moving between the ITM building and O’s South building. The project was conducted as part of the AIDOR (AI and Data Oriented Robotics Service; robot/Internet of Things demonstration support) program operated by the Soft Industry Plaza TEQS, Osaka Industrial Bureau.

### Sensor Deployment and Data Collection

**Figure 1.**
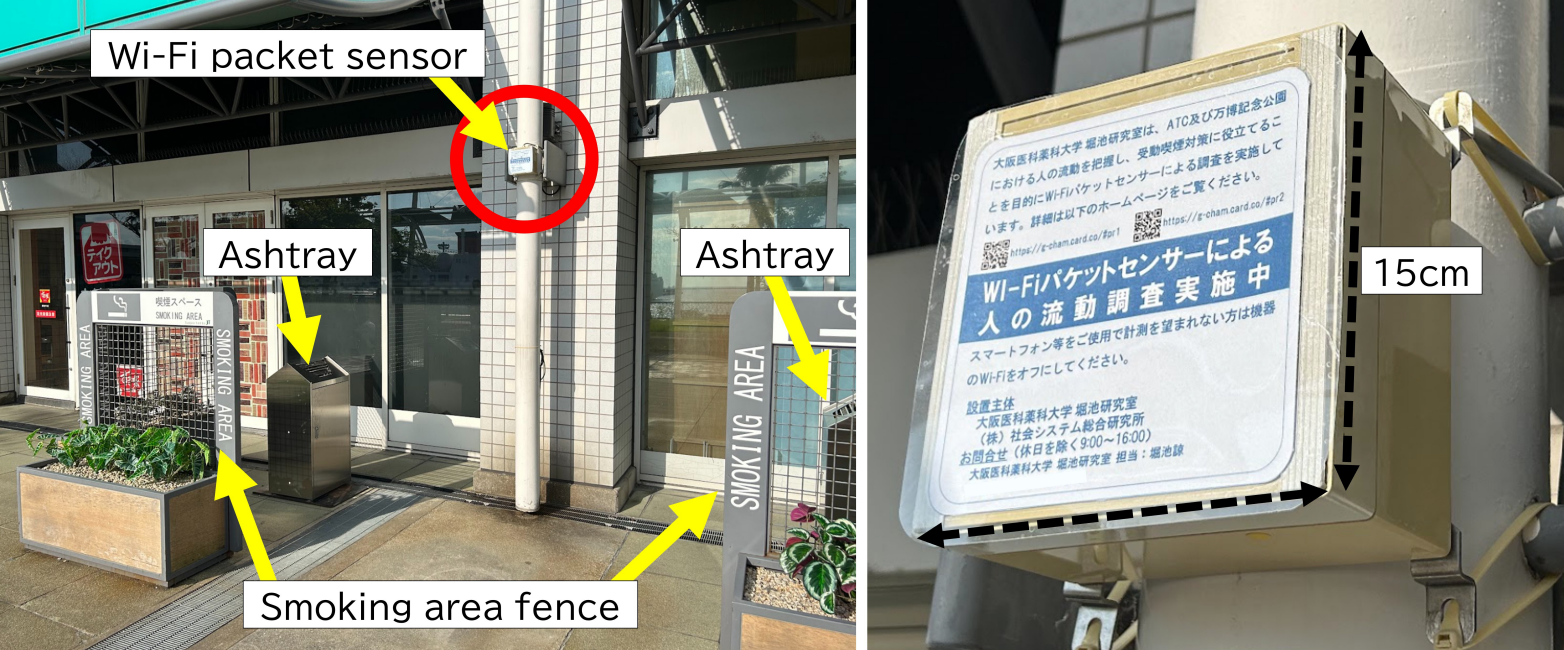
Sensor placement at the Asia Pacific Trade Center outdoor smoking area.

A passive Wi-Fi packet sensor manufactured by Japan Research Institute for Social Systems Co, Ltd was installed with line of sight to both the smoking area perimeter and the adjacent pedestrian corridor (Figure 1). The exact model number and detailed product specifications of the device used in this study were not publicly disclosed. The sensor was mounted on a column at a height of 2.4 m above ground, and the maximum straight-line distance from the sensor to the smoking area fence was 3.15 m. Sensor installation and field operation were conducted with the cooperation of Japan Research Institute for Social Systems Co, Ltd.

The sensor operated in passive monitor mode and collected timestamps, anonymized device identifiers derived from hashed MAC addresses, organizationally unique identifiers, and received signal strength indicator (RSSI) values. The system did not establish network connections with nearby devices and did not collect communication content. Continuous data collection was performed from October 13 to 29, 2023. However, the main analytic dataset was restricted to October 28, 2023. This date was selected because it coincided with Sakishima Kodomo EXPO 2023 and substantial visitor traffic was anticipated. Facility management also indicated that pedestrian traffic was typically sparse on nonevent days. Therefore, October 28 was considered the most appropriate day for calibration and day-level estimation in a high-footfall setting, whereas data collected on the other days were used to confirm normal sensor operation throughout the measurement period.

### Exposure Radius and Validation Window

A circular assessment zone with a radius of 25 meters centered on the smoking area was defined to represent a plausible range within which passersby could encounter measurable outdoor SHS under open-air conditions at this site. This radius was selected a priori for 2 reasons. First, the site geometry and unobstructed line of sight allowed visual coverage of the full area during direct observation. Second, previous field studies have shown that outdoor tobacco smoke may remain detectable several meters from an active smoker and may drift beyond the immediate vicinity in outdoor and semiopen environments [5,6,19]. The validation window was defined as 3 PM to 3:30 PM on October 28, 2023.

### Manual Observation for Calibration

During the validation window, a trained observer counted individuals within the predefined 25-m radius. People handling a lit cigarette within the smoking area were classified as smokers. Individuals who moved through the 25-m radius without smoking were classified as passersby. The observation window was synchronized with continuous sensor logging to permit direct calibration of sensor-derived estimates. The 25-m circle used for observation was created in QGIS (QGIS Development Team), and a hand tally counter was used for on-site recording [20].

### Data Preprocessing and Eligibility

Raw logs were screened to exclude corrupted records and detections outside business hours, which were defined as 10 AM to 10 PM. To identify likely fixed infrastructure devices, organizationally unique identifiers that were observed transmitting outside business hours were treated as fixed devices and excluded from analysis. This rule was used because stationary devices were expected to continue transmitting regardless of facility operating hours, whereas the target observations concerned transient human associated devices during business hours.

For people-count estimation, detections were aggregated into unique anonymized device-based presence episodes within the analysis window. To reduce overcounting caused by bursty probe traffic, a dwell-time eligibility screen was applied. Episodes with dwell times of 6 minutes or less were retained for subsequent classification. This threshold was selected as a pragmatic upper bound for short smoking and passing events in the monitored area. A Japanese study of smoking room users at domestic airports reported mean stay durations of 5.6 minutes for men and 6.5 minutes for women, suggesting that smoking-related stays are typically on the order of several minutes [21]. In this study, the 6-minute threshold was treated as a site-specific operational criterion informed by prior empirical evidence and the on-site validation data rather than as a universally established cutoff.

Wi-Fi probe request analytics and device-based counting have been used in prior studies of pedestrian flow in public-space mobility measurement [15-18].

### Exposure Classification

Eligible presence episodes were classified as likely smokers or likely passersby using a rule-based approach combining RSSI and dwell time. RSSI was used as a proxy for behavioral patterns within the monitored area. On the basis of the empirical distribution observed during the validation window, episodes with RSSI values from −46 dBm to −75 dBm were classified as likely smokers, whereas episodes with RSSI values from −76 dBm to −88 dBm were classified as likely passersby.

The classification thresholds were derived from the validation-day signal distribution and from site-specific observational context, including the open geometry of the smoking area, the fixed sensor position, and observed behavioral differences between smokers, who tended to remain close to the smoking area, and passersby, who typically crossed the monitored corridor laterally. In this study, the rule-based approach was the primary estimation framework.

### Estimation and Calibration Procedure

During the validation window, the ratio of manual counts to concurrent sensor-based classified counts yielded class-specific capture ratios for smokers and passerby. These ratios were then applied to classified sensor counts across business hours on October 28 to estimate day-level totals of smokers and passersby within the 25-m assessment zone. Because direct manual validation was available only on October 28, day-level estimation was restricted to that day. To examine the robustness of the rule-based estimates, sensitivity analyses were conducted by varying the dwell-time and RSSI thresholds by 10% to 20%, and the resulting changes in estimated counts were assessed descriptively. This combination of probe request logs and local calibration is consistent with previous work using wireless detections to estimate pedestrian activity in public settings [15].

In addition to the rule-based analysis, an exploratory random forest model was fitted using stay duration, mean RSSI, and RSSI variability as predictors of smoker versus nonsmoker status. This model was used to explore the classification potential of signal-derived features but was not treated as the primary estimation framework. The analysis was performed using R software (version 4.3.3; R Foundation for Statistical Computing) [22].

### Weather Conditions During Validation

Weather conditions during the validation window on October 28 were sunny, with an air temperature of 20.5 °C, wind speed of 5.6 m/s, and no precipitation. These conditions were recorded to provide environmental context for the outdoor observation and signal data.

### Ethical Considerations

This study analyzed passively detected and anonymized wireless signals from mobile devices and did not involve interaction with, identification of, or intervention involving individuals. Accordingly, it did not constitute human subjects research under the Ethical Guidelines for Medical and Biological Research Involving Human Subjects in Japan [23]. The applicability of the national ethical guideline was reviewed at Osaka Medical and Pharmaceutical University, the authors’ affiliated institution at the time of data collection. The institutional ethics review committee confirmed that the study fell outside the scope of the guideline; therefore, formal ethics approval and informed consent were not required.

No personally identifiable information was collected. Original device identifiers were not retained. Instead, device level records were anonymized via 1-way hashing at the point of capture, and no communication content or network association data were recorded. Data were therefore handled in nonidentifiable form throughout the study. The data collection protocol satisfied the public notice and disclosure items described in the Ministry of Internal Affairs and Communications report on the use of anonymized mobile device signals, including notice to users regarding data acquisition and its purpose at the study site [24].

Sensor placement and short-duration on-site observation were conducted with permission from facility management. No participant compensation was applicable.

## Results

### Overview

On the event day (October 28, 2023), in the validation sample, the Wi-Fi packet sensor recorded 128,313 anonymized MAC addresses. After excluding detections outside business hours (10 AM to 10 PM), 90.3% (115,950/128,313) detections remained. Of these 8.6% (n=11,068) anonymized device identifiers were observed more than once and were therefore eligible for dwell-time estimation. Dwell time could be calculated for 1.4% (n=1817) identifiers, and 0.5% (n=659) presence episodes remained after application of the dwell-time eligibility criterion.

**Figure 2.**
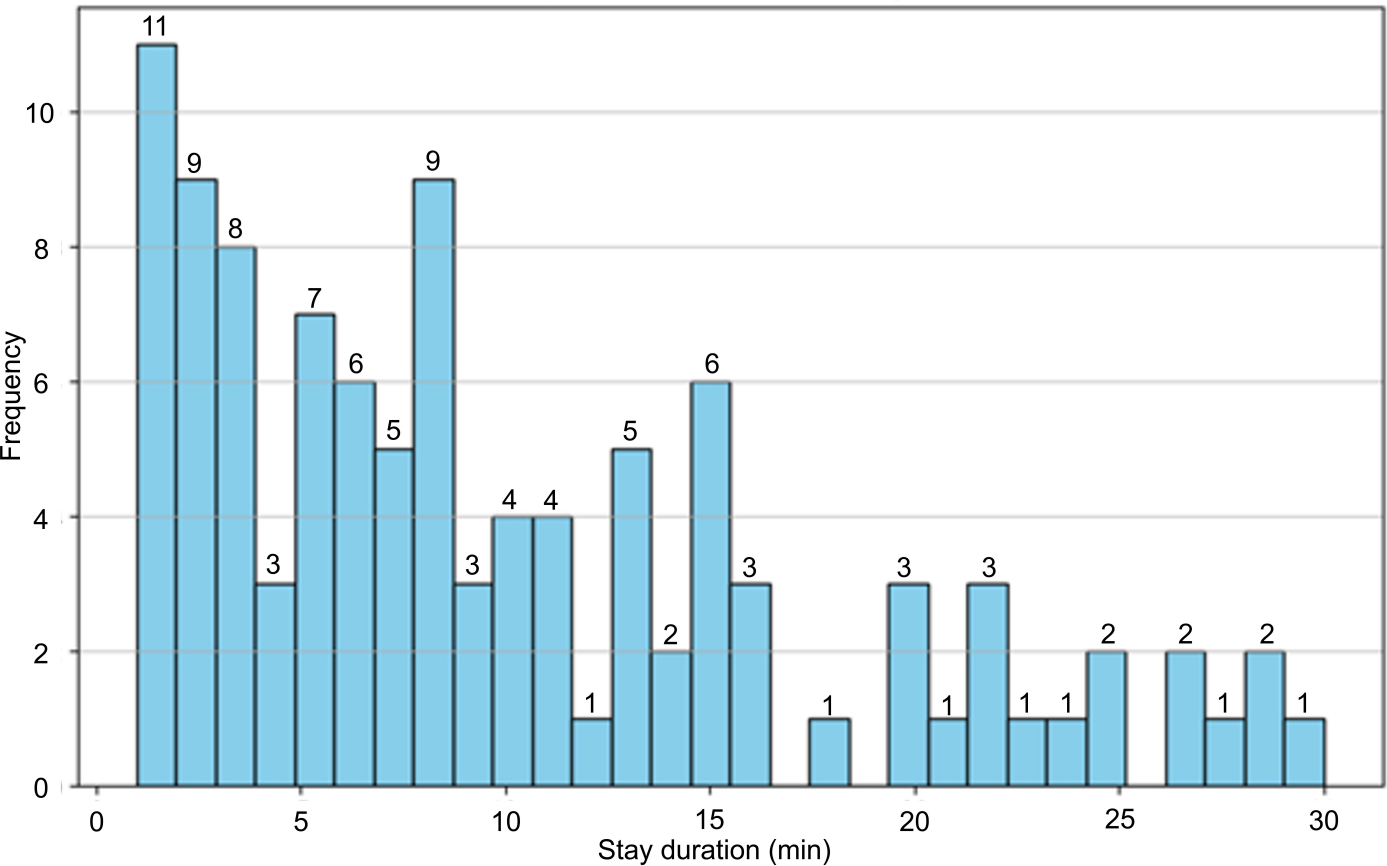
Histogram of stay duration and validation window (3 PM-3:30 PM).

During the validation window, direct observation within the 25-m radius recorded 14 smokers and 207 passersby between 3 PM and 3:30 PM on October 28, 2023. In the same interval, the sensor recorded 6230 signal records, which were aggregated into 104 stays. The mean stay duration was 9.89 (SD 7.89) minutes (Figure 2).

Based on the empirical signal distribution during the validation window, eligible episodes were classified using an RSSI range of −46 dBm to −75 dBm for likely smokers and −76 dBm to −88 dBm for likely passersby. During the validation window, the corresponding capture ratio was approximately 50% (7/14) for smokers and 13.5% (28/207) for passersby. The corresponding device to person ratio was approximately 2.0 for smokers and 7.4 for passersby. The relatively high device to person ratio among passersby was considered plausible because the event was child oriented and many children were unlikely to carry personal mobile devices.

Applying the class-specific capture ratios to business hours data on October 28 yielded estimated day totals of 262 smokers and 3907 passersby within the 25-m assessment zone (Table 1). Temporal aggregation showed bimodal peaks in estimated smoker counts, around noon and 4 PM, whereas estimated passerby volume peaked around noon. Estimated passerby traffic exceeded 500 persons per hour from 11 AM to 4 PM.

**Table 1.** Estimated totals within 25 meters on October 28 (business hours).

Class	Day total (persons), n	Basis
Smokers	262	Classification thresholds + class-specific capture ratio^a^
Passersby within SHS^b^ range	3907	Same as above

a Capture ratios derived from the 3 PM-3:30 PM visual calibration.

b SHS: secondhand smoke.

### Exploratory RSSI-Based Classification Model

**Figure 3.**
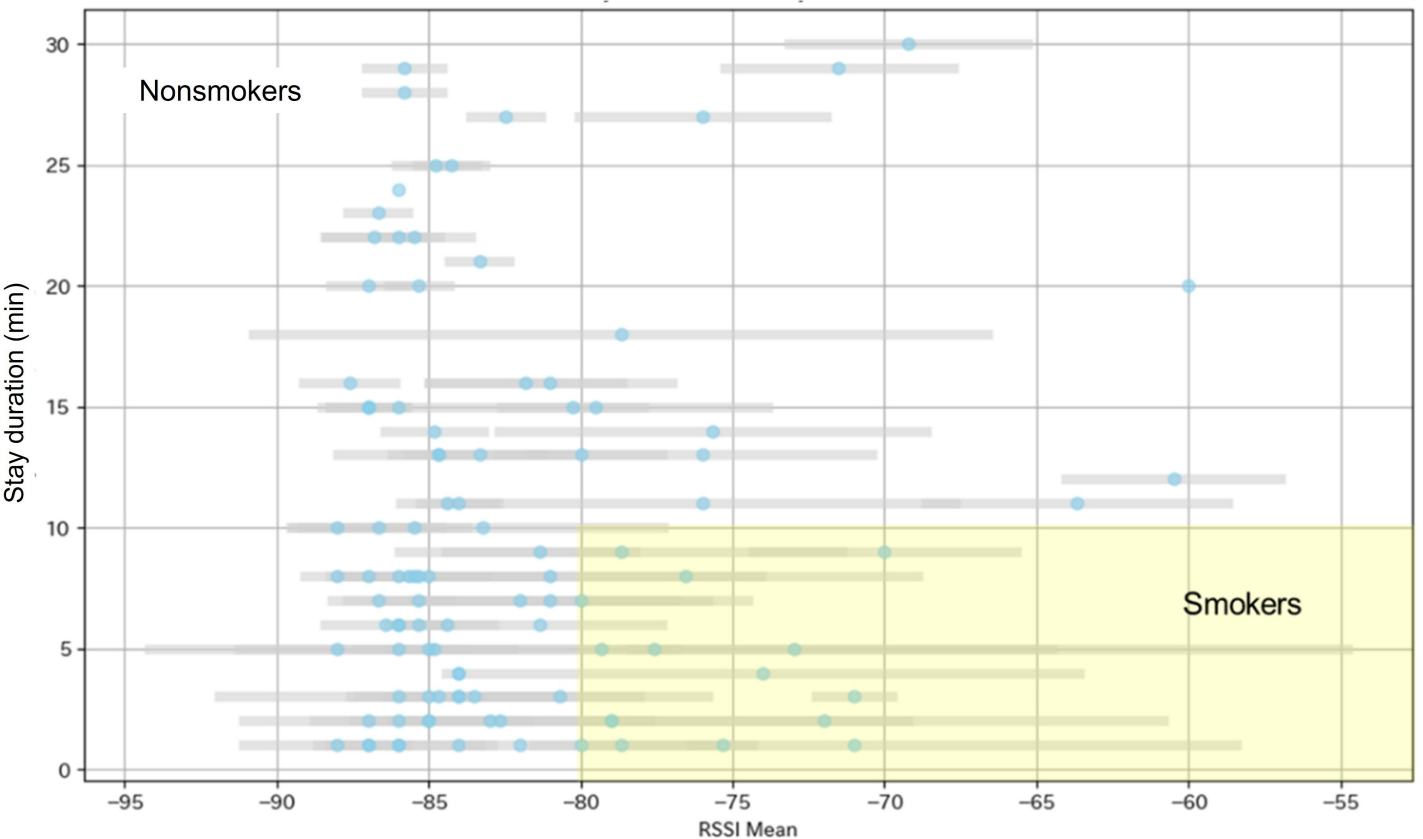
Received signal strength indicator (RSSI) distribution of anonymized device signals during validation window.

As an exploratory analysis, the validation window data were also examined using an RSSI-based classification rule and a random forest model. Using the rule of mean RSSI greater than −80 dBm and stay duration shorter than 10 minutes, 14 stays were identified as smokers, consistent with the number of smokers observed directly (Figure 3).

**Figure 4.**
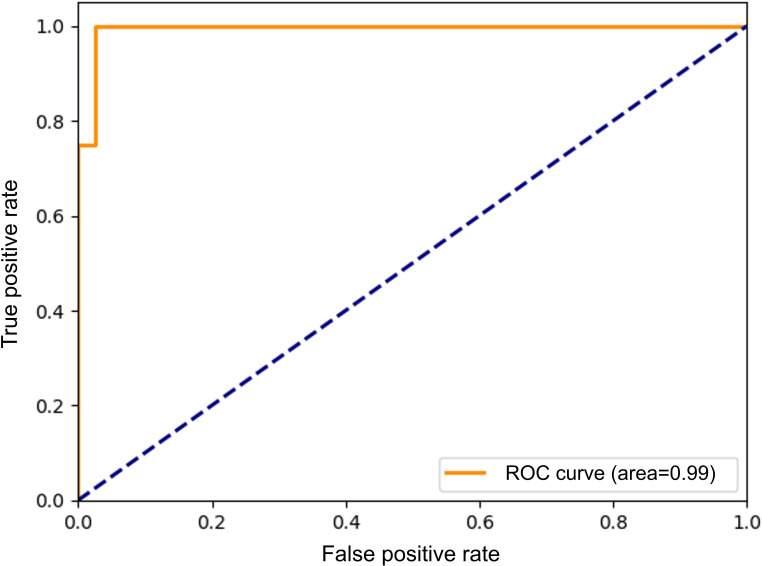
Receiver operating characteristic (ROC) curve of the exploratory random forest classification model.

The exploratory random forest model used stay duration, mean RSSI and RSSI variability to classify smoker versus nonsmoker stays. In the test dataset, the model achieved an accuracy of 0.95, sensitivity of 0.75, specificity of 0.97, and area under the receiver operating characteristic curve of 0.99 (Table 2; Figure 4). The weighted mean precision, recall, and *F*_1_-score were each 0.95. Class-specific precision, recall, and *F*_1_-score were 0.97 for nonsmoker stays (n=38) and 0.75 for smoker stays (n=4).

**Table 2.** Performance of the exploratory random forest classification model^a^^,b^.

	Precision	Recall	*F*_1_-score
Nonsmoker (n=38)	0.97	0.97	0.97
Smoker (n=4)	0.75	0.75	0.75
Weighted average (n=42)	0.95	0.95	0.95

a Best model hyperparameters: n_estimators=10, max_depth=5, and min_samples_split=2; 5-fold cross-validation.

b Overall model performance: accuracy 0.95, sensitivity 0.75, specificity 0.97, and area under the receiver operating characteristic curve (AUC) 0.99.

## Discussion

### Principal Findings

This formative field study demonstrates the practical feasibility of combining passive Wi-Fi packet sensing with brief on-site observation to estimate the numbers of smokers and passersby within a plausible SHS exposure zone around a real-world outdoor smoking area. On a high-footfall event day, at a large commercial complex in Osaka, we estimated 262 smokers and 3907 passersby within a 25-m radius during business hours. These findings suggest that outdoor smoking areas located adjacent to major pedestrian corridors may generate substantial exposure opportunity for nonsmokers, even in settings where indoor smoke-free policies are in place.

The primary contribution of this study is not the direct estimation of inhaled dose, but the operationalization of an exposure-opportunity metric that quantifies how many people may be present within a plausible SHS-affected zone in a real public setting. Previous studies have generally focused either on environmental concentrations or on self-reported exposure at the population level. By contrast, this study addresses a site-specific and operationally relevant question: how many people may be present within a plausible exposure range around a designated outdoor smoking area during routine facility operation? In this sense, this study complements, rather than replaces, concentration-based and survey-based SHS research.

### Interpretation in the Context of Recent SHS and Policy Research

This findings should be interpreted against the broader evidence that SHS remains harmful even at relatively short durations of exposure. Experimental and review studies have shown acute vascular and endothelial effects of passive smoking, and more recent meta-analysis evidence continues to support associations between SHS and multiple cardiovascular outcomes [1-3]. The significance of this literature for this study is that even transient exposure opportunities may be relevant from a public health perspective, particularly when large numbers of passersby are potentially affected over the course of a day.

A substantial literature has also shown that SHS in outdoor and semiopen settings is not negligible. Field studies and reviews of outdoor smoking environments, especially in hospitality settings and partially enclosed spaces, have reported elevated PM2.5 concentrations, smoke drift into adjacent areas, and air quality levels that may fail to meet public health guidance [4-7]. Additional observational studies have documented substantial outdoor SHS exposure in public environments, including settings frequented by minors, reinforcing concerns about routine exposure outside fully enclosed indoor spaces [8-10,25]. This study extends this line of work by indicating that the concern is not only whether SHS is detectable outdoors but also how many people may be present within the affected zone.

At the population level, previous studies have shown that SHS exposure is socially patterned. Recent survey analyses demonstrated that SHS exposure varies by education, employment, income, and other sociodemographic characteristics, suggesting that exposure is not equally distributed across populations [11,12]. However, these studies generally rely on self-reported exposure or broad population indicators and do not quantify how many people are physically present within the exposure zone of a specific outdoor smoking area. Our findings therefore add a complementary perspective: not who tends to report SHS exposure in general, but how many people may be present in a plausible SHS-affected space around a specific smoking area at a given site and time.

The findings are also relevant to the current policy context. Evaluations from Japan indicate that the 2020 revision of the Health Promotion Act, while important, has not fully eliminated SHS exposure and that stronger protections remain warranted [13,14]. This interpretation is also supported by recent Japanese evidence showing that, even after enforcement of the revised Health Promotion Act, many restaurants and bars remained smoking-permitted or noncompliant, highlighting the limitations of partial bans and their enforcement [13,26]. In Europe, evidence from the EUREST-PLUS ITC Europe Surveys likewise showed that SHS exposure in public places remains sensitive to the strength and enforcement of smoke-free legislation, with reductions varying across countries with different legislative regimes [27]. Recent Japanese evidence specifically evaluating SHS exposure around outdoor smoking areas after the partial revision of the Health Promotion Act further underscores that this remains an active and policy-relevant issue in the domestic context [28]. Taken together, these studies suggest that partial protections and designated smoking spaces do not necessarily eliminate SHS exposure opportunity in public settings.

### Methodological Implications and Limitations

This study also contributes methodologically. Passive crowd sensing using wireless probe requests has been widely applied in mobility and pedestrian flow research, where anonymized detections are used to estimate the temporal and spatial distribution of people in public environments [15-18,29]. However, its application to tobacco control and exposure assessment has remained limited. In this study, passive Wi-Fi sensing enabled continuous, noncontact, privacy-preserving observation around a smoking area, and synchronized manual observation allowed the conversion of device-based detections into person-based estimates. The methodological contribution of this study is therefore not the development of a universal exposure model, but the demonstration that such a model can be constructed and operationalized in a real-world setting using modest infrastructure.

At the same time, the study has important limitations. First, the analysis was restricted to a single site and a single event day. Although this was an intentional design choice, given that October 28 was the only day on which direct calibration was conducted and pedestrian traffic was expected to be substantial, the resulting estimates are inherently site-specific and should not be generalized without caution. Second, the estimation framework relied on a single 30-minute manual observation window for calibration, and no additional manual observations were conducted at other times of day or on other days. Accordingly, the calibration ratios used for day-level estimation should be regarded as provisional and context dependent rather than fully validated across temporal conditions. This limitation is particularly important and should temper the interpretation of the estimated day-level counts.

Third, the rule-based classification framework depended on RSSI and dwell time as proxies for proximity and behavior. Both variables are imperfect. RSSI is influenced not only by distance but also by device carriage, body position, surrounding structures, and signal interference, while the dwell-time threshold was adopted as a site-specific operational criterion rather than a universally validated cutoff. The exploratory random forest analysis suggested that signal-derived features may have discriminatory value, but it was based on a small validation sample and should not be interpreted as a definitive or transportable classification model.

Fourth, this study quantified exposure opportunity rather than direct inhaled dose. We did not measure PM2.5, nicotine, or other environmental markers concurrently during the main analysis. Therefore, the results should not be interpreted as evidence of actual individual dose or health effects at the observed site. This is a central limitation of this work and should be addressed in future studies.

### Implications for Practice and Future Research

Future research should integrate environmental monitoring with passive sensing so that exposure counts can be linked to pollutant concentrations and expressed as person-time–weighted or dose-relevant metrics. This would directly address the concern that a more direct exposure model is preferable to a binary classification framework. Such integration may be especially informative in semiopen or wind-affected environments, where SHS concentrations can vary markedly across microlocations [7]. Future work should also include repeated manual validation across multiple time periods, crowd compositions, and sites, especially because the relationship between device counts and people counts is likely to vary according to age structure, device ownership, operating system behavior, and event context.

The practical implications of this study are nonetheless important. If a relatively simple and privacy-preserving sensing system can identify outdoor smoking areas with high exposure opportunity, this information can support decisions about where smoking areas should be located, whether they should be relocated away from major pedestrian corridors or child-oriented spaces, and how smoking area design or surrounding flow management might reduce potential exposure. In this respect, this method may be useful not only for research but also for implementation-oriented public health practice. These findings are also consistent with the World Health Organization Framework Convention on Tobacco Control Article 8 guidelines, which emphasize that effective protection from SHS requires comprehensive smoke-free environments and that partial measures are insufficient where people may continue to be exposed [30,31].

### Conclusions

This formative field study suggests that passive Wi-Fi packet sensing, combined with brief on-site calibration, can be used to estimate the number of smokers and passersby present within a plausible SHS exposure zone around an outdoor smoking area. At a high-footfall public site, the method identified substantial exposure opportunity for nonsmokers, indicating that outdoor smoking areas located near major pedestrian corridors may remain a relevant source of SHS exposure even when indoor smoke-free measures are in place.

The findings should be interpreted as site-specific and preliminary. Nonetheless, they show that exposure-count metrics derived from passive sensing may complement concentration-based and survey-based SHS research by adding a practical estimate of how many people may be present within an affected area. Further studies incorporating repeated validation, direct pollutant monitoring, and multiple sites will be necessary to refine the method and strengthen its usefulness for tobacco control and public health decision-making.

## Abbreviations

AIDOR: AI and Data Oriented Robotics Service
RSSI: received signal strength indicator
SHS: secondhand smoke
